# Potential Residual Contaminants in Edible Bird's Nest

**DOI:** 10.3389/fphar.2021.631136

**Published:** 2021-03-23

**Authors:** Bee-Hui Yeo, Teck-Kim Tang, Shew-Fung Wong, Chin-Ping Tan, Yong Wang, Ling-Zhi Cheong, Oi-Ming Lai

**Affiliations:** ^1^ Institute of Bioscience, Universiti Putra Malaysia, Serdang, Malaysia; ^2^ School of Medicine, International Medical University, Centre for Environmental and Population Health, Institute for Research, Development and Innovation (IRDI), International Medical University, Kuala Lumpur, Malaysia; ^3^ International Joint Laboratory on Plant Oils Processing and Safety (POPS) JNU-UPM, Department of Food Technology, Faculty of Food Science and Technology, Universiti Putra Malaysia, Serdang, Malaysia; ^4^ International Joint Laboratory on Plant Oils Processing and Safety (POPS) JNU-UPM, Department of Food Science and Engineering, College of Science and Engineering, Jinan University, Guangzhou, China; ^5^ Department of Food Science and Engineering, College of Food and Pharmaceutical Sciences, Ningbo University, Ningbo, China; ^6^ Department of Bioprocess Technology, Faculty of Biotechnology & Biomolecular Sciences, Universiti Putra Malaysia, Serdang, Malaysia

**Keywords:** edible bird's nest, nitrite level, heavy metal, color changes, bacteria, fungi

## Abstract

Edible bird’s nest (EBN) is recognized as a nourishing food among Chinese people. The efficacy of EBN was stated in the records of traditional Chinese medicine and its activities have been reported in many researches. Malaysia is the second largest exporter of EBNs in the world, after Indonesia. For many years, EBN trade to China was not regulated until August 2011, when a safety alert was triggered for the consumption of EBNs. China banned the import of EBNs from Malaysia and Indonesia due to high level of nitrite. Since then, the Malaysia government has formulated Malaysia Standards for swiftlet farming (MS 2273:2012), edible bird’s nest processing plant design and management (MS 2333:2010), and edible bird’s nest product quality (MS 2334:2011) to enable the industry to meet the specified standards for the export to China. On the other hand, Indonesia's EBN industry formulated a standard operating procedure (SOP) for exportation to China. Both countries can export EBNs to China by complying with the standards and SOPs. EBN contaminants may include but not limited to nitrite, heavy metals, excessive minerals, fungi, bacteria, and mites. The possible source of contaminants may come from the swiftlet farms and the swiftlets or introduced during processing, storage, and transportation of EBNs, or adulterants. Swiftlet house design and management, and EBN processing affect the bird’s nest color. Degradation of its optical quality has an impact on the selling price, and color changes are tied together with nitrite level. In this review, the current and future prospects of EBNs in Malaysia and Indonesia in terms of their quality, and the research on the contaminants and their effects on EBN color changes are discussed.

## Introduction

Bird’s nest is built by swiftlet using secretions from its salivary glands under the tongue. Nesting can be used as a shelter for canaries to breed and roost. There are more than 24 species of insectivorous, echolocation swiftlets all over the world (Hamzah et al., 2013). However, only two species of swiftlets are responsible for producing commercially valuable edible bird’s nest (EBN) currently. They are species of the *Apodidae*, *Collocalia*, i.e., *Aerodramus fuciphagus* (white nest swiftlets) and *Aerodramus maximus* (black nest swiftlets). EBNs are nests made from the regurgitated saliva. During reproduction, mucin glycoproteins are secreted in saliva from a pair of unique sublingual glands beneath the swiftlet’s tongue, repeatedly interwoven together to build the half bowl shaped, palm size, white nests (Shim and Lee, 2018). When fresh, the nested cement (glutinous secretion/mucin glycoprotein) is soft and viscous, but gradually dries and hardens when exposed to air (Lim and Cranbrook, 2002). EBNs from *A*. *fuciphagus* are favored by processors and traders because they are predominantly hardened nest-like cement with traces of feathers and impurities (Seow et al., 2016), while bird’s nest from others with more feathers and impurities. EBNs are mainly produced in Southeast Asia.

Swiftlets may come from the swiftlet’s house/cave in the morning and forage in the wild like on grass, rice fields, hills, rivers or waters, valleys and trees. Swiftlets do not and never live in any place other than nests, so as to avoid direct interaction or contact with other birds or poultry on land. After looking for food, usually in the afternoon or evening (before dark), the swiftlets return to their own nests. They only return to swiftlet house/cave to rest and lay eggs. Some swiftlets are kept in swiftlet house designed to resemble cave conditions. Swiftlets cannot be cultivated because there is no substitute for food and swiftlets are very dependent on their nature environment. If disturbed or undernourished, swiftlets may not return again to their own nests (Badan Karantina Pertanian, 2013). The diet of swiftlets are mainly insects. The swiftlets perform all their activity in flight, including mating and eating, because swiftlets cannot perch. The EBN in the farmhouse (called the house EBN) is a nest built in the man-made swiftlet house that mimics the cave structure. These swiftlets are attracted by the recorded swiftlet sounds and build nests in the swiftlet house. On the other hand, the cave EBN is the edible bird’s nest in the cave, built by the swiftlet from the natural nesting activities (Quek et al., 2015).

For centuries, EBN is well-known in the Chinese community for its nutritional and medicinal values, is an important ethnomedicinal commodity. The first consumption of EBN dated back to the Tang Dynasty (618–907 AD), when it was the supreme delicacy and was sent to the court of the Chinese emperor. Since then, the medicinal value of EBN has been well documented, and it was later recognized as one of the four major supplements in the late Ming (1405–1433 AD) and early Qing (AD 1644–1911 AD) Dynasties (Chye et al., 2017). EBN is believed to have nourishing effects, such as strengthening the immune system, treating malnutrition, improving metabolism, enhancing skin complexion, relieving asthma, helping to clear sputum, reducing coughs, nourishing children, improving libido, enhancing kidney function, recovery from diseases and surgery rehabilitation, and improving concentration (Tong et al., 2020).

The beneficial properties of EBN have also been proven through modern science and technology, revealing its nutritional values and pharmacological activities, including 1) body maintenance and enhancement of the immune system (Marcone, 2005; Ma and Liu, 2012); 2) stimulation of cell growth (Kong et al., 1987); 3) anti-inflammatory effect (Vimala et al., 2012); 4) protection from joint degeneration and chondro-protection against osteoarthritis (Matsukawa et al., 2011; Chua et al., 2013); 5) enhancement of antioxidant capacity-antioxidative effect (Hu et al., 2016); 6) anti-influenza or as anti-viral agent (Guo et al., 2006; Haghani et al., 2016; Hu et al., 2016); 7) skin whitening, anti-aging, anti-inflammatory and wound healing (Zeng and Lai, 2019; Hwang et al., 2020); 8) promotion of corneal wound healing (eye caring) (Zainal Abidin et al., 2011); 9) improvement of stem cell proliferation (Roh et al., 2012); 10) learning and memory functions of multi-generational mice - (Haghani et al., 2016; Careena et al., 2018; Xie et al., 2018; Khalid et al., 2019; Mahaq et al., 2020); 11) neuroprotection in Alzheimer’s or Parkinson’s disease (Hou et al., 2017; Yew et al., 2018); 12) anti-obesity effects (Yida et al., 2015); 13) prevention of cardio-metabolic and diabetic diseases (Hou et al., 2015); 14) anti-hypertensive effect (Ramachandran et al., 2018); and 15) amelioration of the detrimental effects of lead acetate (LA) toxicity in the uterus (Albishtue et al., 2019). Therefore, EBN has proved its nutritional and therapeutic values.

Raw uncleaned (RUC) EBNs are EBNs harvested from caves and ranches (swiftlet house) which may include sorting, drying, grading, trimming, weighing and packing without any cleaning process and raw-cleaned (RC) EBNs are EBNs that have undergone cleaning processes such as sorting, soaking, picking of feathers and impurities, molding, drying, grading and packing (Department of Standards of Malaysia, 2010). The RC EBN products can be classified as bird’s nest cup (largely maintains its original shape), bird’s nest strip, bird’s nest coner/head (hard ends on both sides of original RUC EBN), bird’s nest crumble (fragments and net of EBN that were collected after processing and packed together), and bird’s nest cake/biscuit (in which EBN fragments or strips or combination are packed into a mold of any shape) (Xing et al., 2012). Traditionally, RC EBN purchased from shop is cooked by double boiling method. The bird's nest, rock sugar and water are placed in an inner pot, and the inner pot is immersed in an outer pot at about 100°C. The stewed product is the bird’s nest soup.

The World Health Organization reports that an estimated 600 million people (approximately one tenth) of the world get sick after eating contaminated food each year, and 420,000 people die each year, resulting in 33 million healthy life years. The WHO also emphasized that nutrition and food safety are inseparable. Unsafe food can lead to a vicious circle of disease and malnutrition, especially affecting infants, young children, the elderly and the sick (WHO, 2020a). EBN is nutritious as listed above, but what about the safe consumption of EBN? This review summarizes previous studies on potential residual contaminants in EBNs, including 1) issues and regulations regarding EBN; 1) nitrite and nitrate content in EBN; 2) the color of EBN; 3) bacteria, fungi and mites in EBN; 4) allergens in EBN; and 5) heavy metals and excessive mineral contents in EBNs and including different stages of EBN, i.e., RUC EBN, RC EBN and EBN after treatment.

## Issue and Regulations Regarding EBN

China is the largest importer of raw clean (RC) EBN products, accounting for 82% of global trade (Zhang et al., 2020). The bird’s nest industry in Malaysia and Indonesia suffered a severe blow in 2011 when China banned exporters’ EBNs due to high concentrations of nitrate, lead and arsenic in certain products. The price of raw uncleaned EBNs plummeted by 50% (Ramalingam, 2014). In 2014, bird’s nest products could be exported to China again. EBN products exported from Malaysia and Indonesia need to be cleaned in accordance with standard operating procedures to reach the EBN safety level required by China. The industry still suffers huge economic losses, and the price of bird’s nest remained stable after 2016 (Chan et al., 2018).

Subsequent to the ban, Malaysia and Indonesia had a few bilateral discussions with the Chinese Authorities and on the April 24, 2012, a Memorandum of understanding (MOU) on the Protocol of Inspection, Quarantine and Hygiene Requirements for the Importation of Bird Nest Products from Indonesia into China was sealed and signed (Badan Karantina Pertanian, 2018); the protocol was sealed and signed between Malaysia and Indonesia on September 19, 2012 (Rohaizan, 2017). Every RC EBN exported to China must comply with this protocol to ensure food safety.

In order to meet the requirements listed in the protocol, Indonesia stipulates that every EBN exported to China should have a traceability system, free from avian influenza, and its nitrite content should be less than 30 ppm (Yusuf et al., 2020). Here is the guidelines provided by Indonesia which can be obtained from the website under Ministry of Agriculture Indonesia government - https://karantina.pertanian.go.id/media.php?module=home: 1) “Pedoman Persyaratan dan Tindakan Karantina Hewan Terhadap Sarang Walet dari Wilayah Negara Republik Indonesia ke Republik Rakyat Cina”- Guidelines for Animal Quarantine Requirements and Measures on EBN, including the traceability system; 2) “Pedoman Pemantauan Karantina Terhadap Pengeluaran Sarang Walet dari Wilayah Negara Republik Indonesia ke Republik Rakyat Tiongkok” - Quarantine and monitoring guidelines for the export of bird’s nest from the territory of the Republic of Indonesia to the People’s Republic of China; 3) “Pedoman Pemanasan Sarang Walet untuk Pengeluaran ke Negara Republik Rakyat Tiongkok” – Guidelines for Heat Treatment of EBN for Expenditure to the People’s Republic of China, to ensure free from avian influenza; 4) “Pedoman Pemeriksaan Kandungan Nitrit Sarang Walet untuk Pengeluaran ke Negara Republik Rakyat Tiongkok” - Guidelines for Examination of EBN Nest Nitrite Content for Exportation to the People's Republic of China, to make sure the nitrite content is less than 30 ppm.

For Malaysia, the requirement listed in the protocol to China also included requirement of traceability system, free from avian influenza and the nitrite content <30 ppm. The quality of RC EBNs from Malaysia for export to China is set by Department of Standard Malaysia as Malaysia Standard. These Malaysia standards include: 1) MS 2333:2010 Good Manufacturing Practice (GMP) For Processing Raw-Unclean and Raw-Clean Edible-Birdnest (EBN); 2) MS2334:2011 Edible- Birdnest (EBN)- Specification; 3) MS 2612:2015 Raw-Unclean Edible Birdnest (EBN)- House nest- Specification; and 4) MS 2509:2012 (P) Test method for Edible-birdnest (EBN)- Determination for nitrite (NO_2_^−^) and nitrate (NO_3_^−^) content. It is worth noting that, besides the above standards, the Malaysia standards also provides standards for farming EBN’s swiftlet, i.e., MS 2273:2012 Good Animal Husbandry Practice - Edible-nest Swiftlet ranching and its premises. MS 2273: 2012 and MS 2333: 2010 are the two important references for EBN industry, inside the standards including a few guidelines to reduce the potential contamination in EBN. Where MS 2273:2012 has showed a few guidelines in ranching practices of edible-nest swiftlet, including the ranch design and maintenance; hygiene of premises; and sign of illness of swifltet. On the other hand MS 2333: 2010 has showed guidelines in designing the processing premises to avoid cross-contamination; control procedure of operation; premise and personal hygiene; and hygiene control system (control contaminant). Those guidelines can reduce the heavy metal, nitrite and microorganisms content in EBNs. Malaysia standard can be purchased from the Department of Standards Malaysia (http://www.standardsmalaysia.gov.my) or SIRIM Berhad (http://sirim.my). Standards and Industrial Research Institute of Malaysia (SIRIM) and Department of Veterinary Service Malaysia (DVS) have published a guideline which is freely available: SIRIM/DVS 2:2014 Requirements for Traceability of Raw Edible- Birdnest (EBN). The Food Safety and Quality Division (FSQD), Ministry of Health (MOH) is the Competent Authority that establishes food safety over the EBN products supply chain in order to ensure that the edible bird’s nest products produced will be safe for human consumption by complying to the Food Act 1983 and Food Regulations 1985, Food Hygiene Regulations 2009 as well as the importing countries’ requirements. FSQD has published a few reference documents: 1) Compliance Listing and Verification Protocol for Export of Raw Clean EBN To China; 2) Standard Operating Procedure (SOP) on the Control of the Safety of Raw EBN Along the Food Supply Chain; 3) SOP on the Control of Nitrite Level in EBN; 4) SOP for Monitoring of Raw Clean EBN; and 5) SOP for Issuance of Health Certificate for the Export of Raw Clean EBN To China. These regulations are implemented and enforced by the Department of Veterinary Services of the Ministry of Agriculture and Food Safety and Quality Division of the Ministry of Health, Malaysia. For meeting the export requirements, an important step is that the EBN products must be processed with heat treatment; the core temperature of the products shall be lower than 70°C and retained at least 3.5 s to effectively kill avian influenza virus.

As an importing country, the Chinese authorities (Chinese Academy of Inspection and Quarantine, CAIQ) have issued two documents on the production of RC EBNs. They are 1) CAIQ-RZ-2015001 Bird’s Nest Product Processing Enterprise, Hygienic Technical Specifications; and 2) CAIQ-RZ-2015002 Bird’s Nest Product Certification Implementation Rules. These two documents can be obtained from the website http://ebn.caiq.org.cn/. The content of CAIQ-RZ-2015001 includes the guidelines for RUC EBNs to RC EBNs processing controls and processing premises while the content of CAIQ-RZ-2015002 includes the guidelines on requirements for the procedures and management of bird's nest product certification activities carried out by certification agencies and this includes the standard of RC EBNs. Table 1 shows the quality of RC EBNs (house nest only) from Malaysia and Indonesia for the export to China. The tolerance levels of different parameters associated with RC EBNs are obtained from MS 2334:2011 for Malaysia Standard; Pedoman Persyaratan dan Tindakan Karantina Hewan Terhadap Sarang Walet dari Wilayah Negara Republik Indonesia ke Republik Rakyat Cina for Indonesia Standard; and CAIQ- RZ-20052 for China Standard. The methods for testing for the parameters were not mentioned in detail for all the parameters. Only heavy metals were mentioned in MS 2334:2011 where AOAC Atomic Absorption Spectrophotometer (AAS) method was used; and nitrite method mentioned in Indonesia Standard uses spectrophotometry or high performance liquid chromatography (HPLC).

**TABLE 1:** Malaysia and Indonesia Standards RC EBNs for export to China.

Category	Parameters	Tolerance level		
		Malaysia	Indonesia	China
Physical	Feather and dirt contamination	N/A	Looks clear of hair and visual debris from the naked eyes at distance of 20–30 cm	N/A
	Metal and wood contamination	N/A	Nil from the naked eyes at distance of 20–30 cm	N/A
Microbiology	Total Plate Count	≤2.5 × 10^6^ cfu/g	≤1 × 10^6^ cfu/g	≤1 × 10^6^ cfu/g
	Coliforms	≤1,100 MPN/g	≤100 cfu/g	≤100 cfu/g
	*Escherichia coli*	≤100 MPN/g	≤10 cfu/g	N/A
	*Salmonella sp.*	Nil	Nil	Nil
	*Staphylococcus aureus*	≤100 MPN/g	≤100 cfu/g	≤100 cfu/g
	Yeast and mold	≤10 cfu/g	N/A	≤10 cfu/g
Residue	Nitrite	≤30 ppm	≤30 ppm	≤30 ppm
	Hydrogen peroxide	Nil	N/A	N/A
	Sulfur dioxide	N/A	N/A	Nil
Heavy metal	Lead (Pb)	≤2 ppm	N/A	≤2 ppm
	Arsenic (As)	≤1 ppm	N/A	≤1 ppm
	Mercury (Hg)	≤0.05 ppm	N/A	≤0.05 ppm
	Cadmium (Cd)	≤1 ppm	N/A	≤1 ppm
Excessive mineral	Copper (Cu)	≤1 ppm	N/A	N/A
	Iron (Fe)	≤0.3 ppm	N/A	N/A

## Nitrate and Nitrite Content in EBN

In August 2011, the Chinese government banned EBN products imported from overseas because of the high levels of nitrite (NO_2_) detected in these edible bird’s nest products. The highest content of nitrite reached 11,000 ppm (cave EBN). According to the report of the Chinese government in Zhejiang Province, the discovery of nitrite pollution in 2011 has aroused public concerns about the safety of EBN consumption. It also aroused the public’s suspicion whether these edible EBNs are really “edible” (Paydar et al., 2013; Quek et al., 2015; Chan et al., 2018).

Nitrate (NO_3_) consists of one nitrogen atom and three oxygen atoms; while nitrite (NO_2_) consists of one nitrogen atom and two oxygen atoms. Nitrite and nitrate are natural chemicals in our food and water. Nitrate is relatively inert and stable, it is unlikely to change and cause harm. Nitrite may become: nitric oxide, which is good for the body; or nitrosamines, which may be harmful. Nearly all manufacturers add nitrite to the meat to protect them, where nitrite is used as a food additive and a preservative, however, the usage is under a strict regulation. In meat, nitrite is converted to nitric oxide. This will react with the protein in the meat, change its color and preserve the meat (Gunnars, 2020).

The daily intake of nitrite acceptable to the World Health Organization is 0–3.7 mg/kg body weight per day or 222 mg/day for 60 kg adults. In the body, nitrite can be converted into nitric oxide (signaling molecule) that can cause blood vessels to dilate and lower blood pressure. When nitrite and amino acids coexist, carcinogenic compounds called nitrosamines are formed during high-temperature cooking (Gunnars, 2020). In order to obtain optimal cardiovascular health and consider potential negative health risks of nitrate and nitrite intake in the diet, foods containing nitrate and nitrite should be guided by reasonable diet (Bedale et al., 2016) and the daily intake limit must not be exceeded.

Table 2 shows the nitrite data from relevant literatures. The data include nitrite from raw uncleaned (RUC), raw cleaned (RC, after processing), house nest, cave nest, different color EBNs and from different parts of EBNs. The table shows that most of the nitrite in white RC house EBNs is less than 30 ppm but not the RC cave nests. The cave nest has a higher nitrite concentration than the house nest. Even after processing, the data shows that the nitrite level of most cave nests is still higher than the allowable level (30 ppm). After processing, the nitrite level of RC EBN is significantly lower than that of RUC EBN, but it is worth paying attention to how effective the processing is for different parts of EBN. Sirenden et al. (2019) showed that the nitrite in different parts of the same EBN is different after processing and suggested that the thickness of each part of the bird’s nest and also the area of the contact surface during processing affects the decrease in nitrite levels.

**TABLE 2:** Nitrite and nitrate levels in different colors of EBNs.

NO	References	Color of EBNs (visual observation description/measurement)	Nitrite (ppm)	Nitrate (ppm)	Source of sample
1	Chan (2013); Chan et al. (2013)	Red	600 (median)		RC: 48 randomly purchased cubilose from Hong Kong market. 25 White EBN, 6 Yellow EBN, and 17 Red EBN. All of these EBN samples were imported from Indonesia, Malaysia, Thailand and Vietnam
		Yellow	510 (median)		
		White	100 (median)		
2	Hamzah et al. (2013)		28.4 (RUC) 0.4 (RC)	349.3 (RUC) 1.2 (RC)	Cave nest from Langkawi, Malaysia
			10.2 (RUC) 0.2 (RC)		Java, Indonesia
			8.5 (RUC) 0.5 (RC)		Balikpapan, Indonesia
3	Paydar et al. (2013)	White	7.9, 12.9, 22	20.4, 23.7, 87.5	RC: Three house-EBNs, Malaysia
		Brown	47.44, 212.9	12,168.2, 2128.6	RC: Two brown cave nests, Southeast Asia
		Red	65, 39.2	30,016.7, 30,016.7	RC: Two red cave nests, Southeast Asia
5	Quek et al. (2015)	Off white to ivory L^*^ value: 50.7 ± 1.7 a* value: 2.1 ± 0.2 B* value: 15.3 ± 0.8	5.7 ± 6.7	98.2 ± 33.7	RC EBN: Four house nests as from Segamat, Johor; Kapar, Selangor; Nibong Tebal, Penang and Sarikei, Sarawak. Cleaned in lab
		Light brown L* value: 37.2 ± 0.8 a* value: 3.0 ± 0.4 B* value: 13.6 ± 0.5	843.8 ± 460.9	36,999.4 ± 38,738.7	RC EBN: Four cave nests as from Gua Gomantong, Sabah; Gua Niah, Sarawak (2 samples); and Gua Subis, Sarawak. Cleaned in lab
6	Susilo et al. (2016)	White	93.12 ± 4.4		RUC EBN : Kalimantan Selatan, Indonesia
7	Quek et al. (2018)	L* value: 50.43 ± 1.84 a* value: 2.08 ± 0.22 B* value: 15.83 ± 1.27	31.63 ± 54.99	133.43 ± 79.22	RUC: Five house nests, four from Peninsular Malaysia, one from East Malaysia. All are *A. fuciphagus* nests
		L* value: 42.19 ± 7.45 a* value: 4.09 ± 1.73 B* value: 17.30 ± 5.47	702 ± 473	31,992 ± 29,569	RUC: Six cave nests, All from East Malaysia. Two samples are *A. fuciphagus* nests and four samples are *A. maximus* nests
8	Sirenden et al. (2019)	White	7.8 (body of EBN)		RC EBN: PT. Waleta Asia Jaya. Bahan baku dari Kecamatan Sepaku, Kabupaten Penajam Paser Utara, Kalimantan Timur
			4.8 (nest of EBN)		
			17.4 (head of EBN)		
9	Yusuf et al. (2020)		32.4, 66.5 and 47.9		RUC EBN: Wajo Regency, South Sulawesi, Indonesia
			7.6, 4.8, and 23.6		RUC EBN: Pare-pare Regency, South Sulawesi, Indonesia
			164.9, 48.8, 136.8		RUC EBN: Pinrang regency, South Sulawesi, Indonesia
10	Tan et al. (2020)	Whitish	10.1 ± 0.4	24.9 ± 0.5	RC: Alor Setar, Malaysia
			10.4 ± 0.2	39.4 ± 1.0	RC: Sibu, Sarawak
			18.4 ± 0.4	52.6 ± 0.9	RC: Rompin, Pahang, Malaysia
			15.8 ± 0.1	47.0 ± 0.6	RC: Kuala Selangor, Selangor
			11.0 ± 0.2	41.5 ± 0.5	RC: Johor Bahru, Johor
			10.3 ± 0.1	31.1 ± 0.5	RC: Jerantut, Pahang, Malaysia
			11.4 ± 0.2	35.9 ± 0.1	RC: Port Klang, Selangor, Malaysia

The results also showed that the nitrate concentration of both house nests and cave nests is always much higher than that of nitrite. Quek et al. (2015) suggested that this situation is due to the stability of nitrate, and nitrate may also be produced by the oxidation of nitrite. Chan et al. (2013) showed that the nitrite content of EBN has a huge range, from non-detectable to 6,430 ppm. Table 2 shows the median concentration of each color of EBNs reported in the study.

Although the nitrite and nitrate in the cave EBNs are significantly higher than the house EBNs on average, it can be seen from the Table 2 that the readings between the samples are widely distributed and the standard deviation value is high, especially in the cave EBNs. These nitrite and nitrate changes may be attributed to different cave and swiftlet farm environments, such as humidity, pH and climate; age of EBN when harvesting (harvesting time); contamination during harvest and the cleaning processes of the collected EBNs may all cause the different concentrations of nitrate and nitrate levels (Paydar et al., 2013; Tan et al., 2020). The good management of the swiftlet houses, such as the frequent removal of swiftlet guano while the guano of the cave is left in the cave and not cleaned may contribute to lower nitrite level in the house nests. The guano can produce nitrite through fermentation (to be discussed later). In addition, good ventilation design of the swiftlet houses also helps to reduce the bacterial anaerobic fermentation process, thereby reducing the concentration of nitrite (Quek et al., 2015; Tan et al., 2020). Therefore, the management and design of the swiftlet houses results in a relatively low concentration of nitrite and nitrate in the house nests compared to the cave nests.

Generally, in any swiftlet house or cave, the presence of nitrite and nitrate is a natural phenomenon. Paydar et al. (2013) hypothesized that the sources of nitrite and nitrate can be obtained from ammonia through anaerobic fermentation by the bacteria. Nitrite is produced by the nest itself and is also absorbed by the swiftlet nesting environment, especially from the floor where organic matter is decomposed. Quek et al. (2015) agreed with Paydar et al. (2013) that the fermentation process of bird droppings and natural environmental resources such as atmosphere, water and soil have caused the infiltration of nitrite and nitrate in bird’s nests.

Chan and his team researched the source of nitrite contamination. In order to find the source of nitrite, they collected swiftlet droppings and water samples from EBN production sites in Malaysia and Indonesia. The results showed that they contained a lot of nitrate instead of nitrite. They also performed proteomic analysis of EBN protein extracts by mass spectrometry. The analysis identified microbial nitrate reductase, which converts nitrate in EBN to nitrite. In EBN, the nitrate/nitrite metabolism process may occur. Under the enzymatic conversion of nitrate reductase, a large amount of nitrate that may originate from swiftlet is triggered to form nitrite. A specific nitrate reductase inhibitor, when added to the EBN under development, can successfully eliminate the nitrate reducing activity found in EBN, thereby reducing the final content of nitrite in EBN. They have successfully proved this possibility in their published study. Therefore, the nitrite on EBN may be the result of environmental pollution and nitrate and microbial nitrate reductase (Chan 2013; Chan et al., 2013; Chan et al., 2018). Swiftlet dropping/guano is source of nitrite content in EBNs, MS 2273:2012 has suggested that farmers should apply Effective Microorganism (EM) on the guano and mine the guano frequently and not allow it to build up.

The nitrite content in bird’s nests should be strictly controlled to ensure that bird’s nests can be eaten safely. According to Malaysia Standard MS2334:2011, raw cleaned edible bird’s nests (RC EBN) which after undergone cleaning processes should not contain more than 30 ppm of nitrite. A proper cleaning process can reduce the nitrite and nitrate concentrations in EBNs (Chan et al., 2013; Hamzah et al., 2013). There are only limited studies (Quek et al., 2018; Tan et al., 2020) comparing the levels of nitrite and nitrate in house/farmed EBNs from different regions. Most studies compared between houses and cave/wild EBNs. In addition, the comparative study of nitrite and nitrate of different RC EBN products (such as cup-shaped EBN, instant cook, etc.); and how different treatment methods affect the content of nitrite have yet to be reported.

## The Color of EBN

When customers choose EBNs, color is an important attribute and indicator of food quality and food acceptability. The color of house EBN is usually white, off-white, light yellow, brown, golden yellow and orange red. Majority of the house EBNs are off white and light yellow. On the other hand, the color of cave EBNs is usually white but with "red head," red, orange-red and brown. Majority of cave nests are red and orange in color. In fact, all EBNs start from white. After 2–3 months, the EBN color changes from white to yellow. After about 6 months, when RUC EBN is still in ranches, the EBNs finally turn red (Quek et al., 2015). Unlike cave EBNs, not all house EBNs will eventually turn red after a long period of time.

According to ancient traditional Chinese medicine, white EBN is mainly used to treat coughs and other respiratory diseases, and only red EBNs can be used to treat children with dysentery i.e., blood in the stool (Chan et al., 2013). The supply of red EBNs or cave EBNs is very limited. Higher demand than the supply leads to higher price of red EBNs than white EBNs. The high nutritional and medicinal values and the higher price of the red EBNs lead to issues of adulteration (Ma and Liu, 2012). Marcone (2005) mentioned that on occasion white nests have been treated with red pigments which are either partially or wholly water-soluble; and But et al. (2013) reported that white nest was fumigated with “bird soil” under hot and humid condition. After treatment of the white EBNs, falsified appearance as red EBN will be sold as higher grade and athigher price.

The bird’s nest is initially white, but the color may remain white or change to other colors during harvest. Different colors of EBN are initially secreted through the beaks of the same species of swiftlet, so they are not different due to structural coloration (Shim and Lee, 2018). The causes of color changes has been a puzzle for centuries. The following shows the process and reasons for the color change of bird's nest from the previous studies. But et al. (2013) showed that white EBNs could turn yellowish/red/darker red by vapors from bird soil. The white EBNs turn yellowish when “washed bird soil” was used for fumigating the EBNs. The white EBNs turn red (lighter) when “untreated bird soil “was used for fumigating the EBNs. The darker red EBNs are observed, when the white EBNs were fumigated with “heated bird soil” (bird soil placed in oven for 5 h) and “nitrite-enriched bird soil” (“wash bird soil” mixed with sodium nitrite). The study has also shown that the vapors produced by sodium nitrite (NaNO_2_) under acidic conditions (2% HCl) can turn white EBNs into red, while the vapors produced by NaNO_2_ in distilled water cannot (But et al., 2013). Thus, swiftlet excrement can be a source for changing the color of raw uncleaned bird nests.

Paydar et al. (2013) showed two important findings; i.e., 1) no hemoglobin was detected in red “blood” nests; and 2) nitrite and nitrate in EBN affect EBN’s color. No hemoglobin was detected in red “blood” nest or cave nest and this answers to an old folklore which asserted that the red EBNs are swiftlet blood mixed with saliva when exhausted swiftlets hurried to finish their nests before laying eggs. Paydar et al. (2013) also found that when white EBNs were exposed to vapor from NaNO_2_ in 2% HCl, or bird soil turn to brown/red colors, where nitrite and nitrate contents also increased significantly. This results agree with the comparison between house (white) nests and cave (red) nests, where cave nests (red/brown) contained higher intensities of C-N and N-O bonds compared to house nests. However, Payder et al. found that nitrite and nitrate are not the only factor that affect the reddening process, because some brown EBNs nitrite and nitrate are higher than red EBNs; and white EBNs soaked into the NaNO_2_ in 2% HCl and exposed to vapor from bird soil nitrite enriched bird soil turned to yellowish and not turned to red (Paydar et al., 2013). Chan (2013)’s finding is consistent with Paydar et al. (2013)’s finding, where white and yellow EBNs turned into red after 10–20 days of incubation with 1M potassium nitrate. On the other hand, no significant color change occurred for the samples without nitrate sources. A strong positive correlation between nitrite with redness (a* color component) and negative correlation with lightness (L* color component) were found with Pearson correlation (Quek et al., 2015).

Shim and Lee (2018) reported that the white nests turned to red/orange color because of the vapor of reactive nitrogen species reacting with the mucin glycoprotein tyrosine. This reaction is named as nitration of tyrosine. The reactive nitrogen species used in the study is nitrous acid (HNO). In the bird house or cave, after aerobic bacteria (nitrifying bacteria) decompose bird soil, they produce nitrous acid and nitric acid vapor. Shim and Lee showed that the nitration of the tyrosine residues gave 3-nitrotyrosine residue in the glycoprotein (3-NTyr) and caused the red color. Tyrosine has been proposed as one of the markers for differentiating between house and cave EBNs, as house EBN has higher tyrosine compare with cave EBN (Seow et al., 2016). This is due to the fact that tyrosine in white EBN is not nitrated.

Wong Z. C. F. et al. (2018) suggested that EBN has potential metallic binding sites, where they found that white EBN was changed to red with the addition of Fe^3+^; and changed to blue when Cu^2+^ was added. They proposed that transition metals induce color change in EBNs, and binding of Fe ion to acidic mammalian chitinase-like (AMCase-like) protein directs the origin of red color in EBNs. They proposed that NaNO_2_ can be an oxidant in EBN, through the oxidization of iron ions process, oxygen is bound to Fe and leads to the color change in EBNs where increased in Fe-O bond intensity was found (Wong ZCF. et al., 2018).

Gan and his team in 2016 and 2017 have conducted a series of studies regarding the relationship between rehydration/drying and color change in EBNs. EBN samples were subjected to 1) convection hot air drying at 40, 50, 70, 80, and 90°C, air circulation at 4.6 m/s; 2) intermittent with infrared coupled with ultraviolet C (UVC) at 25 and 40°C, intermittent α = 0.2, 0.33, 0.67 (tempering periods of 240, 120, and 60 min) and α = 1; and 3) heat pump drying intermittent (α = 0.2, 0.33, 0.67, and 1.0) with air velocity at 28.6 and 40.6°C, during “on”: high air velocity at 5.4 ± 0.5 m/s and during “off” low air velocity of 1.0 ± 0.5 m/s. The lowest total color change happened at intermittent α = 0.2 air velocity at 28.6°C; and the highest color change was reported at 90°C hot air drying. Overall, results demonstrated that air velocity or infrared and intermittent UVC contributed to lower color change. Gan and his team suggested that during the tempering period, in the low-temperature dehumidified air in the drying chamber, the moisture from the center of the material will be redistributed to the surface, which is likely to prevent dehydration of the surface and has a significant effect on reducing color changes because of the rate of Maillard reaction which is reduced and non-enzymatic browning. On the other hand, hot air drying method contains high levels of oxygen, which stimulates the enzymatic browning reaction (Gan et al., 2016, 2017).

Zhang et al. (2020) successfully showed that low-energy X-ray can inactivate foodborne pathogen, but the irradiation also affects the color of EBNs. Study showed that the when irradiation dose increases, the yellowness value increases while the lightness decreases respectively. Studies above showed that color changes in EBNs occur when in the swiftlet houses and during the processing from RUC to RC. Based on studies by Paydar et al. (2013), Chan (2013), Shim and Lee (2018) and Wong Z. C. F. et al. (2018), one can conclude that nitrite and nitrate play an important role in EBN color changes. Bird soil could be the most likely sources of the nitrite and nitrate. But the source is not limited to nitrite and nitrate as the metal like Fe ion and high temperature during the drying process can also affect the color changes in EBNs. Here, the studies focused mainly on the color change from white to darker color like yellow, brown and red nests. How about from yellow or brown nests to white nests? Recently, consumers prefer house nests that are white or ivory in color but not yellow or brown nests. To the best of our knowledge, there is no study that focus on the EBN color changes from darker to lighter shades. Semicarbazide (SEM), a potentially harmful agent, was detected in instant bottled bird’s nests, cup-shaped EBN and bird nest cakse. The hypothesized sources of SEM were from edible gum and hypochlorite used for bleaching (Xing et al., 2012). Bleaching is a step that can make the color of EBN become lighter, but until recently there are still no detailed study on the effect of bleaching to the EBNs.

## Bacterial, Fungi and Mites in EBNs

Bacteria associated with EBNs may cause food-borne diseases if ingested. Bacteria enter the body through contaminated food or water. Food-borne pathogens can cause severe diarrhea or debilitating infections such as meningitis (WHO, 2020a). As shown in the Table 1, in the EBN study, several microorganisms were of concerns, namely coliforms, *Escherichia coli* (*E. coli), Salmonella, Staphylococcus aureus*, yeast and mold. Coliform is the “indicative organism” of food microbiology. Coliforms are Gram-negative, rod-shaped, non-spore forming bacteria, such as *Citrobacter*, *Enterobacter, Escherichia,* and *Klebsiella* species (Martin et al., 2016).

*Salmonella* sp. and *E. coli* are the most common food-borne pathogens, affecting millions of people every year, and can be fatal (WHO, 2020a). *Enterobacter* sp. are another coliform that cause nosocomial infections. *Staphylococcus aureus* cause serious threat to human health globally as Staphylococcal food poisoning. Elimination of *Staphylococcus aureus* is very important for food industry, but is very challenging (Fetsch and Johler, 2018). Fungi are the other most resilient spoilage microorganisms. Fungi can overcome the food safety control strategy adopted by the food industry. Fungi can multiply in foods with extremely limited water supply and have extremely high heat resistance as they can survive and thrive in commercial sterilized foods (Snyder and Worobo, 2018). Fungi can appear as yeast, mold or a combination of the two forms. In addition to the controlled microorganisms that affect the quality of EBNs, there are another organisms that might affect the quality of the EBNs such as mites. Mites have been identified as pathogens and are the most common source of allergens that cause respiratory allergies and anaphylaxis. After ingesting heated or unheated mite-contaminated food, systemic anaphylaxis can occur in those who have been sensitized (Sanchez-Borges et al., 1997).

Table 3 shows that mites, fungi and bacteria have been successfully isolated and identified in previous studies while Table 4 shows the concentration of mites, fungi and bacteria that have been reported. Kew et al. (2014) revealed the presence of mites, fungi, bacteria and feathers on both RUC and RC EBNs through structural analysis of EBNs under a scanning electron microscope. Mite egg-shells and fecal particles as well as body parts of other arthropods were found on the RUC EBNs. Bacterial (streptococci, cocci and rod-shaped) and fungal structures (yeast, hyphae and fungal spores) were detected on the surface of RUC EBNs. The mites, bacteria and fungi were also detected on the surface of RC EBNs.

**TABLE 3:** Bacteria, fungi and mites associated with EBNs.

No	References	Type of samples	Microbes	Microbes After treatment
1	Wong et al. (2018a)	Raw uncleaned (house nest)	Bacteria (isolates) *Acinetobacter sp.,Brevibacterium sp., Bacillus subtilis, Bacillus shackletonii, Bacillus sp., Bacillus megaterium, Bacillus pumilus, Bacillus flexus, Bacillus circulans, Bacillus cereus, Bacillus aryabhattai, Deinccoccus sp., Enterococcus faecalis, Enterococcus sp. , Listeria fleischmannii, Microbacterium sp. ,Paenibacillus sp., Paenibacillus sp. 23-13, Paenibacillus agglomerans, Paenibacillus alvei, Staphylococcus nepalensis, Staphylococcus Kloosi, Staphylococcus sp., Staphylococcus sciuri, Staphylococcus sp. Y3 Virgibacillus halophilus*	Double boiling *Bacillus subtilis, Bacillus sp.,*
		Raw cleaned (commercial EBNs)	*Bacteria (isolates) Acinetobacter sp., Acinetobacter radioresistens, Acinetobacter calcoaceticus, Brevibacillus sp. Brevibacterium sp., Bacillus sp., Bacillus badius, Bacillus cereus, Bacillus flexus, Bacillus lichniformis, Caryphanon sp., Deinococcus sp. Enterobacter cloacae, Enterobacter hormaechei Exiguobacterium sp., Solibacillus silvestris Staphylococcus sp., Staphylococcus pasteuri, Staphyloccus saprophyticus, Staphylococcus sciuri. Sporosarcina saromensis*	Double boiling *Brevibacillus sp., Brevibacillus agri, Bacillus sp.*
2	Kew et al. (2015)	Raw and commercial nests	Mites (Isolates) *Eustathia cultrifer*, *Pteroherpus garrulacis*, *Pterodectes amaurochalinus*, *Laminalloptes* sp., *Berlesella alata, Neochauliacia* sp., *Suidasia* sp., *Austroglycyphagus* sp., *Aleuroglyphus ovatus, Dermanyssus sp., Cheyletus sp.,* Tarsonemid, cunaxid mites*, Collocalidectes sp., Streetacarus sp., Hemisarcoptes sp* and unidentified oribatid mites	N/A
3	Sien et al. (2013)	Swiftlet feces in swiftlet farm houses	Bacteria (Isolates) *Bacillus sp., Dermacoccus sp. 103, Enterococcus harae strain ss33b, Escherichia coli, Leucobacter iarius strain 40, Lysinibacillys sp. B4, Paenibacillus sp. Gh-134, Proteus sp., Pseudomonas aeruginosa strain 123, Sporasarcina sp., Staphylococcus sp.*	N/A
4	Sani et al. (2015)	Raw cleaned EBN	Mold (Isolates) *Aspergillus* spp. and *Penicillium* spp.	
5	Chen et al. (2015)	Raw uncleaned (house nest)	Fungi (Isolates) Soil Fungi: *Blastobotrys* sp., *Lichtheimia* sp., *Nigrospora* sp., *Paecilomyces* sp., *Perenniporia* sp., *Phialosimplex* sp. *Syncephalatrum* sp.*, Sagenomella* sp.*, Stephanoascus* sp. *Talaromyces* sp*,* Plant Fungi: *Coprinellus* sp.*, Fomitopsis* sp.*, Lasiodiplodia* sp.*, Lenzites* sp.*, Letendraea* sp.*, Polyporales* sp.*, Rigidoporus* sp. Environmental Fungi: *Aspergillus* sp., *Candida* sp., *Cladosporium* sp., *Neurospora* sp., *Penicillum* sp., *Eurotium* sp.	Double boiling Soil Fungi: *Phialosimplex* sp. Plant Fungi: -Environmental Fungi: *Aspergillus* sp., *Candida* sp., *Cladosporium* sp., *Neurospora* sp., *Penicillum* sp., *Eurotium* sp.
		Raw cleaned (commercial EBNs)	Fungi (Isolates) Soil Fungi: *Chrysosporium* sp., *Nigrospora* sp., *Sagenomella* sp., *Sebanicales* sp. Plant Fungi: *-* Environmental Fungi: *Aspergillus* sp., *Candida* sp., *Cladosporium* sp., *Neurospora* sp., *Penicillum* sp.	

**TABLE 4:** Bacteria, fungi and mites contents in EBNs.

No	References	Type of samples			Enumeration method	Source of samples
		Raw uncleaned EBN	Raw cleaned EBN	After treatment/Others		
1	Tan et al. (2020)		ND *E. coli, S. Aureus* and *Salmonella*		Australian Standard- *Escherichia coli, Samonella spp*., Coliform, and total plate count. Official AOAC method –*Staphylococcus aureus*	RC: Seven RUC house nest samples from different regions in Malaysia then cleaned in lab. Seven regions include: Alor Setar, Kedah; Sibu, Sarawak; Rompin, Pahang; Kuala Selangor; Johor Bahru; Jerantut, Pahang; and Port Klang, Selangor
			Total Plate Count: 2.3*10^5^–25*10^5^ cfu/g			
			Coliform ND- 43 cfu/g			
			Mould <10–140 cfu/g		Bacteriological Analytical Manual of Food and Drug Authority- mould and yeast	
			Yeast <10–10 cfu/g			
2	Wong et al. (2018a)	6.0*10^2^–1.02*10^5^ CFU/0g		*Double boiling* 0–2.4*10^2^ CFU/g	Total Plate Count	RUC: Five Malaysia house nest from Kuala Sanglang, Pantai Remis, Kluang, Kajang and Kota Bharu
			4.0*10^1^–1.5*10^5^ CFU/g	*Double boiling* 0–2.4*10^2^ CFU/g		RC: Six commercial sample purchase from five different Chinese traditional medicine shops from Malaysia and one from Medan, Indonesia
3	Sien et al. (2013)			Swiftlet feces 6.03–9.22 log10 CFU/g	Total Plate Count	Swiftlet feces from swiftlet houses located in ten places, including Kota Samarahan, Saratok, Semarang, Betong, Sarikei, Sibu, Sepinang, Maludam, Kuching and Miri in Sarawak
4	Sani et al. (2015)		7.64–7.66 log CFU/g	*Gamma Irradiation 20 kGy* <2 log CFU/g	Total Plate Count	RC: Raw cleaned samples from Pahang and Terengganu
			5.61–5.95 log CFU/g	Gamma irradiation 5 kGy <2–4.64 log CFU/g	Plate count -agar Brilliance Coliform-Coliforms	
			2.47–2.67 log CFU/g	Gamma irradiation 1 kGy <2 log CFU/g	Plate count agar Brilliance *E. coli-* *E. coli*	
			4.55–4.66 log CFU/g	Gamma irradiation 5 kGy <2 log CFU/g	Plate count – agar Rabbit Plasma Fibrinogen *-* *Staphylococcus aureus*	
			4.8–5.10 log CFU/g	Gamma irradiation 5 kGy <3 log CFU/g	Plate count-agar Dichloran rose Bengal chloramphenicol- Yeast and molds	
			Not detected	N/A	Plate count- agar xylose lysine deoxycholate agar and brilliant green agar- *Salmonella spp*.	
5	Chen et al. (2015)	40–18,080 CFU/g	40–2,640 CFU/g	N/A	Plate count- Sabouraud Detrose Agar- Fungi	RUC and RC: Same sample batch with Wong et al. (2018a) exclude sample from Medan
6	Kew et al. (2015)	Live 0–66.4 mites/g. Dead 15.9–2,613 mites/g. Total 18–2,613 mites/g.	Live 0 mites/g. Dead 0–88 mites/g. Total 0–88 mites/g.	N/A	Stereomicroscope- Mite	RUC and RC: Same sample batch with Wong et al. (2018a) exclude sample from Medan.

Sien et al. (2013) successfully isolated 500 bacterial isolates (11 types, Table 3) from the swiftlet feces collected from a farmhouse in Sarawak, Malaysia. The 16srRNA analysis showed that 96% of the isolates were identified as Gram-positive bacteria, and the remaining 4% of the isolates were Gram-negative bacteria. *Staphylococcus sp.* was the most prevalent bacteria found in feces.

*E. coli, S. aureus* (Sani et al., 2015) and *Enterobacter* (Wong ZCF. et al., 2018) were detected in the RC bird nests. No *Salmonella* was detected by both studies including RUC EBNs. Mold (Sani et al., 2015) and fungi (soil and environmental fungi) (Wong S. F. et al., 2018) were detected in RC EBNs also.

Sani et al. (2015) reported the concentration of total plate count (TPC), coliforms, *E. coli*, *S. aureus*, and yeast and mold before and after irradiation. Before the radiation, RC EBNs readings exceeded the permittable limit set by Standard Malaysia. Similarly, as reported by Chen et al. (2015), all EBNs had fungal CFU that exceeded the limit (100 CFU/g). However, when RUC EBNs and RC EBNs were compared, there were no significant difference found between the total number of bacteria and fungi in both the samples but they did show significant difference in types of isolates (Chen et al., 2015; Wong S. F. et al., 2018).

Wong S. F. et al. (2018) reported that *Enterobacter, Exiguobacterium, Brevibacillus, Caryphanon,* and *Solibacillus* species were found exclusively in the commercial EBNs. RUC and RC EBNs were purchased separately and therefore, the raw materials before processing into RC EBNs might differ from the RUC EBNs. *Bacillus* species were found in all type of samples, including bird’s nest feces, RUC EBN, RC EBN and double boiled samples. *Bacillus cereus* can cause foodborne illness through the production of distinct toxins which lead to diarrhea and emetic syndrome (Griffiths and Schraft, 2017). In addition, the spores of *Bacillus cereus* are highly resistance to processing and harsh conditions, and can germinate to vegetative cells under any favorable conditions (Lv et al., 2019). Therefore, it is recommended that attention should be paid to the existence and quantity of *Bacillus* in the EBN industry.

Plant fungi were only detected in RUC EBN samples, but not RC EBNs and also boiled samples. *Aspergillus* sp. and *Penicillum* sp. are the common fungi isolated from the unboiled and boiled raw (RUC) and commercial (RC) EBNs (Chen et al., 2015; Sani et al., 2015). *Aspergillus* sp. and *Penicillum* sp. are environmental fungi and can be easily isolated from spoiled food. They produce mycotoxins that are harmful to human health (Greeff-Laubscher et al., 2020). This is not only for the safe consumption of EBN products, but also may pose risk of exposure to farmers and workers who deal with or process the raw uncleaned EBNs.

Kew et al. (2015) observed that there were no live mites detected in RC EBNs. This is consistent with the structural analysis, where the mites observed at RC EBNs were partially embedded in the nests. Kew et al. (2015) have successfully isolated and identified thirty types of mites. They suggested that these isolates probably are feather mites, house dust and storage mites, mesostigmatid mites, prostigmatid mites, astigmatid mites and oribatid mites.

Previous studies (Kew et al., 2014; Chen et al., 2015; Wong et al., 2018a) suggested that the bacteria, fungi and mites on the EBNs may be originated from the fauna and detritus found in the cave, swiftlet houses and the surrounding as well as the insects ingested by the swiftlets. Remnants of insects in their mouths and possibly the microbes and mites on their body are incorporated into the nests when the swiftlets build their nests with their saliva. Bacteria, fungi and mites in the houses and/or surrounding the ranches can directly contaminate the EBNs during harvest or contaminate the swiftlets, or inhabit the feathers and skin of swiftlets which indirectly contaminate the EBNs. Fungi and microbes can be introduced during EBN processing, storing and transporting.

From Tables 3, 4, we can observe that double boiling can significantly reduce types and number of the bacteria. However, this method may not be effective in removing heat-resistant bacteria such as *Bacillus sp.* and *Brevibacillus* sp. In addition, double boiling of EBNs was not effective in reducing the types and number of fungi, and this suggested fungi also possessed heat-resistant properties. Gamma radiation was reported to reduce the number of yeast and molds. Other studies have shown that microwave sterilization can reduce the number of *Salmonella* and *E. coli* in EBN drink (Than et al., 2018); and heat sterilization can remove yeast and mold, coliform*, E. coli*, *Salmonella,* and *S. aereus* effectively with no detectable growth in EBN beverages (Lam, 2018). Low-energy X-ray irradiation (350–400 Gy) can decreased *E. coli* O157:H7 and *S. Typhimurium* in dry EBNs from 6.35 to 5.84 log CFU/g, respectively, to undetectable level (Zhang et al., 2020).

## Allergens in EBNs

Food allergy is defined as hypersensitivity reaction towards a food. Food allergic reactions can be classified as immediate (IgE mediated reaction) and delayed (generally non-IgE-mediated reaction) (Kemp et al., 2010). Anaphylaxis is a severe systemic hypersensitivity reaction with rapid onset; characterized by life-threatening airway, breathing, and/or circulatory problems; and usually associated with skin and mucosal changes (Reber et al., 2017). EBN can be a potential source of life-threatening food allergy to those who are sensitized to its components or contaminants. The National University Hospital of Singapore reported that among children, 0–15 years old, the most common food allergen is bird’s nest soup, which surpasses other clear food allergens, such as milk, eggs, peanuts and crustaceans (Goh et al., 1999). The allergic symptoms after consumption of bird’s nest soup were typical of a type I hypersensitivity reaction (angioedema, wheezing, urticaria, and abdominal cramps), and no deaths were reported.

Goh and his team continued to study allergens in EBNs, and they found that IgE-mediated hypersensitivity occurs after consumption of bird nest soup. Investigations revealed that 66 kDa protein is the main putative allergen responsible for this reaction. The protein is homology with ovoinhibitor, Kazal-type serine protease inhibitor, that is mainly found in chicken egg white (Goh et al., 2000; Goh et al., 2001; Ou et al., 2001). One band with a molecular weight of approximately 77 kDA protein found from white and red “blood” nests, this protein that similar to those of the ovotransferrin protein in eggs to the highly allergenic ovotransferrin protein in eggs. This protein can be partially responsible for the allergic reactions (Marcone, 2005).

The source of allergens in EBNs has not been determined, because of the 39 cases of allergic reactions, 14 of them had eaten edible bird’s nest before, without any allergic reactions (Goh et al., 2000). Those who develop allergic reactions after consuming bird's nest soup may be sensitized to the bird nest components or other associated contaminants. Kew et al. (2015) suggested that the possible sources of allergens found in the EBNs may originate from the saliva or feathers of the swiftlets, the insects ingested by the swiftlets, the microorganisms and arthropods (mites) associated or inhabit with the nests or swiftlet, the cleaning processes of the raw nests, the adulterants added to the raw cleaned EBNs and the contaminants introduced, and the infestation of arthropods or other organisms during the storage of the nests. Most of the possible allergens listed above can be removed by proper cleaning process and management after cleaning. However, the heat-resistant bacteria, fungi and mite allergens can be causes of concern.

## Heavy Metals and Excessive Mineral Contents in EBNs

This section summarizes the studies on heavy metals (lead, arsenic, mercury and cadmium) and excessive minerals (iron and copper) in EBNs, and the content of these elements is subjected to the maximum limit of RC EBN in Malaysian standards (Table 1). Metals and metalloids (a combination of metal and non-metal elements) found in foods can be beneficial or harmful when within or exceed the recommended dose (FDA, 2020c). Pb, As, Hg, and Cd are heavy metals that have no established health benefits and are harmful to human (FDA, 2020a). Fe and Cu are beneficial metals to human when taken in optimum dosage and not exceeded. It is discussed here as excessive mineral (as listed in Malaysia Standard).

Lead may be a cumulative toxic substance that affects multiple body systems. Long-term exposure to low concentrations for an extended time may also be dangerous. It is toxic to humans, especially harmful to vulnerable people such as babies, young children, pregnant women and their fetus, and people with chronic diseases. Lead within the body is distributed to the brain, liver, kidneys and bones. It is stored in teeth and bones and accumulates over time. High levels of lead exposure can seriously endanger the health and development of kids, especially the brain and nervous system. It affects learning abilities, causes behavioral difficulties and lowers IQ. Important sources of environmental pollution to lead pollution include mining, smelting, manufacturing and recycling activities, continued used of leaded paints, leaded gasoline and leaded aviation fuel in certain countries/regions. Lead is detected in food due to its presence in the environment and enters our food supply through the following methods: 1) lead within the soil can settle or be absorbed by plants, fruits or vegetables or plant-based dietary supplements; 2) lead in food cannot be removed completely by washing or other food processing steps; 3) The animals we eat may also ingest and absorb lead in plants or water, and pass it on to us; 4) lead may inadvertently enter through the manufacturing process (for example, lead-containing pipes can contaminate water employed in food production); 5) lead in some pottery and other food containers may leach out and enter into food when food is prepared, served or stored (FDA, 2020b; WHO, 2020b).

Arsenic (As) is omnipresent components in environment and humans are exposed to arsenic through air, ground water and food sources. Significant wellsprings of arsenic defilement could be either through geological or anthropogenic activities (Mohammed Abdul et al., 2015). Arsenic in the environment is normally low, except for volcanic emissions, mining, breaking, coal-terminated force plants, arsenic-treated wood, and arsenic-containing pesticide contamination in certain zones. Arsenic enters the food supply through soil, water or air. Arsenic in food can cause life-threatening health problems (FDA, 2020a). Arsenic influences practically all cellular processes and organ functions in our body, like integumentary, cardiovascular and reproductive systems. In addition, arsenic is able to cause epigenetic modifications and tumorigenesis, subsequently causing cancer (Mohammed Abdul et al., 2015).

Mercury can be classified into three main groups: metallic mercury (Hg^0^), inorganic mercury (Hg^2+^), and organic mercury (methyl mercury: CH_3_Hg^+^). Minamata disease is a typical example of the mercury pollution-related health damage (Sakamoto et al., 2018). Mercury has been widely used in many measuring instruments, electric appliances, agricultural chemicals and mildew-proofing agents. Human exposure to mercury occurs through leakage from the instruments and appliances and land run-off of this chemical. Environment may be polluted by mercury through discharge from industrial activities (anthropogenic) or derived from volcanoes (natural) (Clarkson, 1997; Sakamoto et al., 2018). The health effects of mercury include but are not limited to behavioral and cognitive changes; prenatal exposure to methylmercury (MeHg) is associated with low birth weight, delayed neurodevelopment, and growth and development of children; and increased risk of psychiatric symptoms (Clarkson, 1997; Ha et al., 2017).

Cadmium (Cd) is a toxic heavy metal and is a non-essential trace metal that has strong teratogenic and mutagenic effects in living organisms. General population exposure to cadmium is through occupational exposure (example working at metal refinery industry), diet (the main source), breathing, smoking, or drinking water (Fatima et al., 2019; Geng and Wang, 2019). Local residents near an active lead-zinc mine and copper smelter acquired the Cd via dietary intakes of rice and vegetables (Du et al., 2020). The impact of cadmium on human health includes damage to the lungs, glucose impairment which leads to diabetes, restriction of fetal growth, impaired hormone synthesis, kidney diseases (renal failure, renal anemia, etc.), anaemia, osteoporosis, cardiovascular diseases and cancer (Nordberg et al., 2018; Fatima et al., 2019; Geng and Wang, 2019; Kumar and Sharma, 2019).

Copper within the atmosphere is produced by natural processes and human activities. Natural processes include volcanic eruption, windblown dust and microbial activities while human activities include industrial activities such as acid drainage from mining operation. Human exposure to copper is through daily intake, mainly from food like seafood and dry fruits, and water (Latorre et al., 2019). Copper is an important micronutrient in the human body, and it contains about 100 mg of copper in the human body. Copper is involved in a variety of biological processes including antioxidant defense, neuropeptide synthesis and immune function (Bost et al., 2016). However, excess or deficit of copper is linked to several human disorders like diabetes, cardiovascular diseases and cancer. Wilson disease (copper excess) and Menkes disease (copper deficiency) are the two major genetic disorders that have been widely studied in terms of copper homeostasis (Latorre et al., 2019).

Maintaining iron homeostasis in human body is important. Deficiency or over exposure to iron has noticeable effects on human health. Iron is an essential nutrient for human as it participates in a wide variety of metabolic processes, including supporting oxygen binding and transport (e.g., hemoglobin); electron transport (e.g., heme-containing enzymes), oxidative metabolism (e.g., NADH dehydrogenase), cellular proliferation and DNA synthesis. Iron deficiency always manifests as anemia, which results in reduced immunity, reduced work ability and impaired mental function. Maintaining excessive iron in the human body can lead to neurodegenerative diseases, liver damage, endocrine disease, cardiac dysfunction, and increased risk of liver cancer (Abbaspour et al., 2014; Wessling-Resnick, 2017). Therefore, maintaining the homeostasis state of copper and iron in the human body is vital.

Table 5 shows the concentration of heavy metals and excessive minerals detected in EBNs. Studies found that 1) lead, arsenic and cadmium are always below the regulatory limits even for the concentration in RUC EBNs; 2) most of the RUC EBNs had higher mercury and copper levels than the limits; 3) however mercury and copper concentrations in RC EBNs were below the regulatory limits; and 4) all samples had higher iron concentration than the permissible limit of 0.3 ppm. There are very limited studies on heavy metals and minerals in EBNs. From the classification, latent Dirichlet allocation model suggested that arsenic and mercury contributed most significantly (*p* < 0.05) to the geographical origin differentiation (between Peninsular Malaysia and East Malaysia (Quek et al., 2018). Quek et al. (2018) suggested that the difference is due to location of swiftlet houses and caves, where the Peninsular Malaysia is concentrated with many industrial activities. These activities emit toxic metal pollutants that contaminate the environment and ecosystem, including forages for the swiftlets. Swiftlets are aerial insectivores that usually forage for insects around their habitats. Another reason they suggested is due to indoor environments of the swiftlet houses, for example paints used for painting the swiftlet houses could be the source of mercury contamination, pesticides used to control the bacteria and pests in or around the swiftlet houses can also be sources for arsenic and mercury contamination. Research on heavy metals and minerals is very limited, especially on RC EBNs. There is no research reporting on the source of mercury, copper or iron contamination, or whether current cleaning processes are able to remove these contaminants effectively.

**TABLE 5:** Heavy metals and excessive mineral contents in EBNs.

NO	References	Sample	Element concentration (ppm)							Source of sample
			Lead, Pb	Arsenic, As	Mercury, Hg		Cadmium, Cd	Copper, Cu	Iron, Fe	
1	Tan et al. (2020)	House nest	<0.02	<0.01	<0.01	<0.01				RC: Seven RUC house nest samples from different regions in Malaysia then cleaned in lab
2	Quek et al. (2018)	House nest	0.164 ± 0.089	0.069 ± 0.014	0.081 ± 0.007	0.003 ± 0.001		5.22 ± 0.45	21.58 ± 10.56	RUC: Five house nests, four from Peninsular Malaysia, one from East Malaysia. All is *A. fuciphagus* nest
		Cave nest	0.190 ± 0.086	0.067 ± 0.007	0.073 ± 0.005	0.005 ± 0.002		5.13 ± 0.75	23.01 ± 9.05	RUC: Six cave nests, All from East Malaysia. Two samples are *A. fuciphagus* nest and Four samples are *A. maximus* nest
		Peninsular Malaysia	0.184 ± 0.089	0.075 ± 0.007	0.083 ± 0.006	0.003 ± 0.001		5.33 ± 0.44	18.67 ± 9.74	RUC: Four house nests. All is *A. fuciphagus* nest
		East Malaysia	0.174 ± 0.088	0.065 ± 0.010	0.073 ± 0.005	0.005 ± 0.002		5.08 ± 0.67	24.47 ± 9.12	RUC: One house nest and six cave nests. Three samples are *A. fuciphagus* nest and Four samples are *A. maximus* nest
3	Salim et al. (2018)	House nest		<0.05						Received from Ministry of Health, Malaysia
		Cave nest		0.1–0.3						
4	Chen et al. (2014)	RUC: House nest	<0.592	0.000055–0.034.45	0.000060–0.070.180 *1sample >0.05 ppm	0.060–1.870		<0.500	1.309,725–1.3496320	Same sample batch with Wong et al. (2018a) exclude sample from Medan
		RC: Commercial	<0.067	<0.01	Nd- 0.0144			0.19113–0.53883	>0.3 <5.790,405 *All >0.3 ppm	

## Recommendations and Conclusion

This critical review has made an attempt to discuss the most important findings in the understanding of potential residual contaminants in EBNs. A number of knowledge gaps have been identified in each of the potential residual contaminants, including nitrite and nitrate contents; bacteria, fungi and mites; heavy metals; and other contaminants in EBNs and their effects on EBN color changes and allergenicity.

Some of the identified gaps that need to be investigated include:

1. Comparative study of potential residual contaminants in different RC EBN products, such as cup-shaped EBN, instant cook, etc.;
2. Comparative study of different processing (including cleaning, drying and sterilization) methods (from RUC EBN to RC EBN) in removing and mitigating the contaminants;
3. Study on the effect of bleaching as during the cleaning process;
4. Study on the factors which influencing color changes during the drying process.

There also needs to be a close working relationship between researchers and EBN industry players to fill the gap of knowledge. In addition, the policy makers can formulate reasonable and effective policies based on science and “real” industry conditions. The heat-resistant bacteria or fungi and allergens should be included in the RC EBN standards formulated as well. In a nutshell, there is an urgent need for more investigation to gain insights into potential residual contamination of EBNs.
